# Inverse-cavity structure for low-threshold miniature lasers

**DOI:** 10.1038/s41598-022-15319-y

**Published:** 2022-07-05

**Authors:** Gunpyo Kim, Seok Ho Song, Jae Woong Yoon

**Affiliations:** Department of Physics, Hanyang University, Seoul, 04763 Korea

**Keywords:** Semiconductor lasers, Nanophotonics and plasmonics

## Abstract

Creating micro and nano lasers, high threshold gain is an inherent problem that have critically restricted their great technological potentials. Here, we propose an inverse-cavity laser structure where its threshold gain in the shortest-cavity regime is order-of-magnitude lower than the conventional cavity configurations. In the proposed structure, a resonant feedback mechanism efficiently transfers external optical gain to the cavity mode at a higher rate for a shorter cavity, hence resulting in the threshold gain reducing with decreasing cavity length in stark contrast to the conventional cavity structures. We provide a fundamental theory and rigorous numerical analyses confirming the feasibility of the proposed structure. Remarkably, the threshold gain reduces down by a factor ~ 10^−3^ for a vertical-cavity surface-emitting laser structure and ~ 0.17 for a lattice-plasmonic nanocavity structure. Therefore, the proposed approach may produce extremely efficient miniature lasers desirable for variety of applications potentially beyond the present limitations.

## Introduction

Miniature lasers have led to far-reaching technological advances for variety of application areas including telecommunications, data processing, medical detection, and display to mention a few^1–4^. Since the invention of vertical-cavity surface-emitting laser (VCSEL)^5^, extensive study on miniature lasers has been conducted to create various promising structure classes such as whispering-gallery microcavities^6^, photonic crystal defect-mode cavities^7^, and synthetic nanowire resonators^8^. Further miniaturization has been proposed in terms of surface-plasmonic resonance structures to take advantages of sub-diffraction-limited optical confinement and ultrafast dynamics in femtosecond time scales^9–11^.

Along this line, miniaturization of lasers has continuously raised formidable challenges including high threshold gain. Compared with macroscopic lasers, micro and nano lasers require very high material gain in order to compensate remarkably increased radiative losses over a much shorter optical path length. Consequently, desired laser operation often involves extreme conditions^12–14^ in material processing, temperature management, and pump controls even with the most-efficient gain media such as organic dyes^15^ and semiconductors^16^.

In this paper, we propose an inverse-cavity structure for micro or nano laser operation in remarkably low threshold-gain conditions. The proposed inverse-cavity structure consists of a passive cavity enclosed by amplified-feedback mechanisms, which is in an exactly opposite configuration to conventional laser structures with an amplifying cavity and dissipative feedback mechanisms. In this inverse-cavity configuration, modal amplification rate increases with decreasing cavity length as the amplified feedback events occur more frequently in a shorter cavity. Therefore, the proposed cavity structure yields lower threshold gain for a shorter cavity in stark contrast to the conventional cavity configurations. We provide a fundamental theory, efficient method to produce an amplified-feedback effect by means of nanophotonic resonances, and rigorous numerical calculations for experimentally conceivable vertical-cavity surface-emitting laser (VCSEL) and lattice-plasmonic nanocavity structures. Intriguingly, we theoretically obtain threshold-gain reduction from the conventional cavity configuration by a factor 10^−3^ for an AlGaAs VCSEL structure and 0.17 for an Au-InGaAs lattice-plasmonic laser structure in their shortest-cavity conditions. Therefore, our result suggests a promising approach for creating extremely low-threshold miniature laser elements.

## Results

### Fundamental theory

A common basic structure of conventional lasers consists of a leaky resonant cavity containing a gain medium. Such a cavity structure implies minimally conceivable cavity length

1 $$L_{{\text{c}}} = \frac{{\ln \eta_{{\text{f}}}^{ - 1} }}{{2(G_{{{\text{max}}}} - \alpha )}},$$

where *G*_max_ is the maximum modal-gain constant obtainable from the gain medium, *α* is modal dissipation rate, and *η*_f_ is power efficiency of the feedback mechanism for one optical roundtrip inside the cavity. The inverse proportionality between *L*_c_ and *G*_max_ is an essential consequence because the number of dissipative feedback events per unit time and subsequent radiative losses increase as the cavity becomes shorter. Consequently, reducing *L*_c_ to a certain required level for micro and nano lasers in the wavelength or subwavelength scales involves very high *G*_max_ or *η*_f_ at the cost of extreme controls in temperature, pumping density, and precise formation of feedback mechanisms^12–14^.

**Figure 1:**
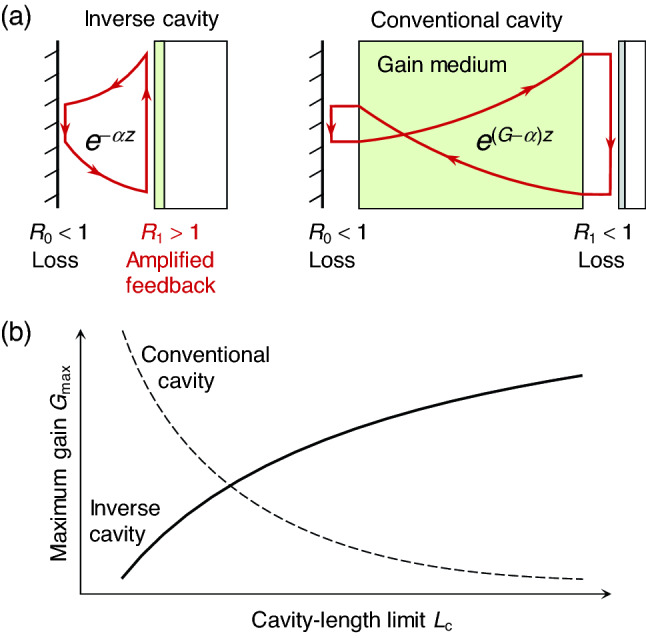
Inverse-cavity concept in comparison with the conventional cavity configuration. (**a**) Structure configuration of an inverse cavity versus a conventional cavity. Arrowed loops indicate instantaneous optical intensity during one roundtrip at lasing threshold. (**b**) Relation between minimal cavity length limit *L*_c_ and maximum gain constant *G*_max_ for an inverse-cavity structure in comparison with a conventional cavity structure.

In search of possible solutions to this inherent problem, we consider an inverse-cavity structure where optical gain is provided by a coherently amplified feedback mechanism instead of an intracavity gain medium, as shown in Fig. 1a in comparison with a conventional cavity configuration. In this inverse-cavity structure, we assume the amplified feedback mechanism provides feedback efficiency *η*_f_ = *R*_0_*R*_1_ > 1 so that it compensates attenuation exp(–2*αL*_c_) during passive intracavity propagation. In stark contrast to the conventional cavity configuration, this inverse-cavity structure implies lower required optical gain for a shorter cavity because the cavity mode acquires higher gain for a shorter cavity by providing more frequent amplified feedback events.

Considering the gain–loss balance for stationary optical oscillation in an inverse-cavity structure, the threshold condition for amplified reflectance *R*_1_ is

2 $$R_{1} = \frac{1}{{R_{0} }}\exp (2\alpha L_{{\text{c}}} ).$$

Since the threshold *R*_1_ exponentially decreases with reducing *L*_c_, the required optical gain *G*_max_ implicitly related to *R*_1_ should also decrease for smaller *L*_c_, as schematically illustrated in Fig. 1b. Therefore, the proposed inverse-cavity structure can be remarkably efficient especially for short-cavity lasers.

**Figure 2:**
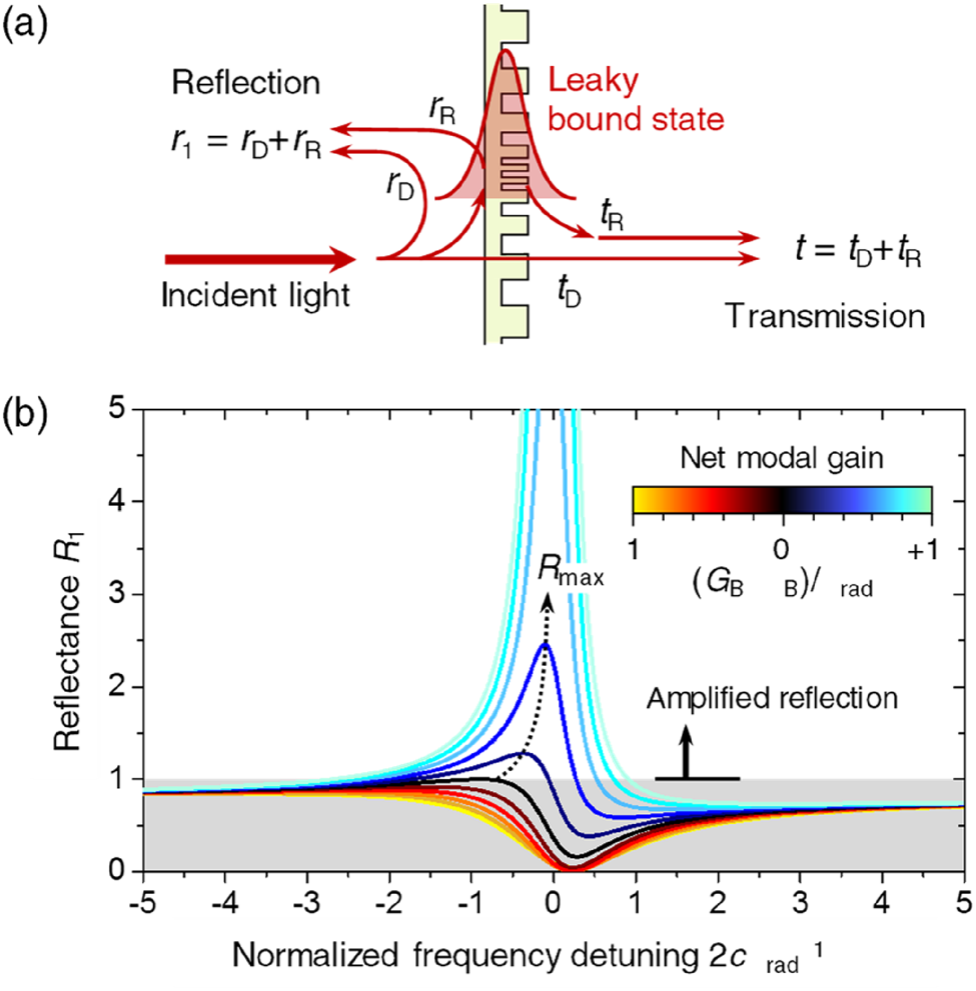
Amplified reflection from a nanophotonic resonance element. (**a**) Schematic illustration of two-channel resonant scattering due to a leaky bound state in a nanophotonic structure. *r*_R_ and *t*_R_ represent coefficients of the resonant reflection and transmission through a leaky bound state while the non-resonant scattering is described by *r*_D_ and *t*_D_. The reflectivity *r*_1_ and transmissivity *t* are coherent superpositions of these resonant and non-resonant pathways. (**b**) Amplified reflection *R*_1_ =|*r*_1_|^2^ =|*r*_D_ + *r*_R_|^2^ spectra for increasing net modal gain *G*_B_–*α*_B_ from –*α*_rad_ to + *α*_rad_, where *G*_B_, *α*_B_, and *α*_rad_ represent modal gain, absorption, and radiative decay constants, respectively. In this calculation, we assume non-resonant reflectance |*r*_D_|^2^ = 0.8, non-resonant transmittance |*t*_D_|^2^ = 1–|*r*_D_|^2^ = 0.2, and radiative-decay probability toward the reflection channel *η*_1_ = 0.7 for a certain presumable example.

The amplified-feedback mechanism is a key component in this inverse-cavity laser concept. It is conveniently obtainable by using resonant optical scattering in various nanophotonic structures. Resonant nanophotonic scattering in many cases can be treated as a two-channel Fano-resonance problem that describes reflection coefficient *r*_1_ as a superposition of non-resonant reflectivity *r*_D_ and resonant reflectivity *r*_R_, as schematically illustrated in Fig. 2a. A generic temporal coupled-mode theory for this two-channel resonance problem^17^ predicts the reflectance (*R*_1_ =|*r*_1_|^2^) spectrum depending on net modal-gain constant, as shown in Fig. 2b. We indicate net modal gain constant *G*_B_–*α*_B_ relative to radiative attenuation constant *α*_rad_, where *G*_B_ and *α*_B_ are modal gain and absorption constant for the leaky bound state. Maximum reflectance *R*_max_ exceeds 1 for *G*_B_–*α*_B_ > 0, indicating the amplified reflection required for the inverse-cavity laser operation.

In particular, *R*_max_ in the small-signal approximation is determined by

3 $$R_{\max } \approx 1 + C_{1} \frac{{G_{{\text{B}}} - \alpha_{{\text{B}}} }}{{\alpha_{{{\text{rad}}}} - (G_{{\text{B}}} - \alpha_{{\text{B}}} )}} + C_{2} \left[ {\frac{{G_{{\text{B}}} - \alpha_{{\text{B}}} }}{{\alpha_{{{\text{rad}}}} - (G_{{\text{B}}} - \alpha_{{\text{B}}} )}}} \right]^{2} ,$$

where dimensionless coefficients *C*_1_ and *C*_2_ are functions of resonance coupling rates, |*r*_D_|, and the phase difference between *r*_R_ and *r*_D_. See Supplementary Note I for details of the derivation based on a geometric representation of Fano-resonance scattering amplitudes. Obviously, the amplified reflection (*R*_max_ > 1) is obtainable in the leaky-bound-state amplification regime for *G*_B_–*α*_B_ > 0. Considering an inverse-cavity laser that employs the amplified reflection from a leaky bound-state resonance in the short cavity (*αL*_c_ <  < 1) and high back-reflection (*R*_0_ ≈ 1) limits, the threshold condition in Eq. (2) combined with Eq. (3) predicts *L*_c_ such that

4 $$L_{{\text{c}}} \approx \alpha^{ - 1} \left[ {C_{1} \frac{{\left( {G_{{\text{B}}} - \alpha_{{\text{B}}} } \right)}}{{\alpha_{{{\text{rad}}}} }} + (C_{1} + C_{2} )\frac{{\left( {G_{{\text{B}}} - \alpha_{{\text{B}}} } \right)^{2} }}{{\alpha_{{{\text{rad}}}}^{2} }}} \right].$$

Therefore, threshold modal-gain constant *G*_B_–*α*_B_ required for an inverse-cavity laser operation decreases as the cavity gets shorter.

Use of a resonant reflector as the amplified feedback mechanism suggests another advantage in the optical coherence of output light because of a doubly resonant arrangement of the structure. The lasing mode in the proposed inverse-cavity structure oscillates in both the mode-selecting cavity and resonant reflector simultaneously. Every feedback event inside the mode-selecting cavity involves resonant excitation of a leaky bound state in the amplifying reflector. Therefore, photon density *N*_R_ in the resonant reflector containing the gain medium is substantially enhanced from photon density *N*_C_ in the mode-selecting cavity such that *N*_R_ ≈ (2π*D*/*λ*)^−1^*Q*_R_⋅*N*_C_, where *D* is thickness of the resonant reflector, *λ* is wavelength of light, and *Q*_R_ is resonance quality factor of the resonant reflector. This implies that the stimulated emission rate in the gain medium is enhanced by a factor ~ (2π*D*/*λ*)^−1^*Q*_R_ from that expected if the gain medium was contained in the mode-selecting cavity. Therefore, an inverse-cavity laser can produce output light with substantially higher coherence than conventional cavity structures with the mode-selecting cavity being in a similar length scale.

### Numerical analysis

In order to see if the proposed inverse-cavity structure produces remarkably low-threshold laser oscillation in practical systems, we perform rigorous numerical analyses on two distinct nanophotonic cavity structures. They are VCSEL and lattice-plasmonic cavity structures.

**Figure 3:**
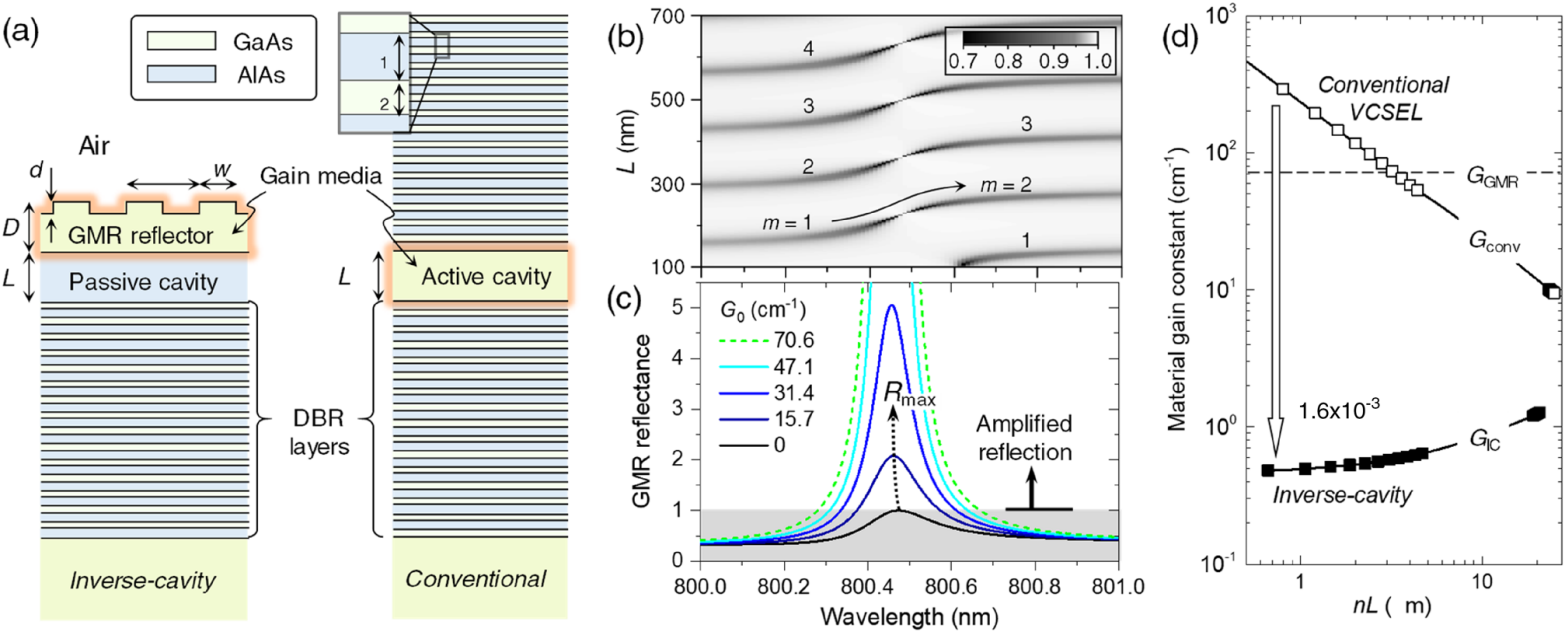
VCSEL employing an inverse-cavity structure. (**a**) Geometry of a GaAs-AlAs-based inverse-cavity structure in comparison with a conventional VCSEL structure. Optical gain is applied to the GaAs guided-mode-resonance (GMR) reflector for the inverse-cavity structure and GaAs Fabry-Pérot cavity for the conventional VCSEL structure. (**b**) Reflectance spectrum on *L*-*λ* plane for an inverse-cavity structure under normal incidence from the air cover. Geometrical parameters and optical constants are *d* = 25 nm, *D* = 475 nm, *Λ* = 226.5 nm, *w* = *Λ*/2, *δ*_1_ = 67.8 nm, *δ*_2_ = 55.6 nm, and refractive indices 2.95 for AlAs and 3.6 for GaAs. Material gain constant *G*_0_ = 0 in the GaAs GMR reflector for this spectrum and *m* denotes a Fabry-Pérot (FP) order. (**c**) Amplified internal reflectance spectra due to a gain-assisted GMR for increasing *G*_0_. (**d**) Calculated threshold gain constants *G*_IC_ for the inverse-cavity structure and *G*_conv_ for the conventional VCSEL structure as functions of cavity optical path length *nL*. *G*_GMR_ is threshold gain constant for the GMR in the absence of the bottom DBR layers (*L* = ∞).

In the VCSEL structure study, we compare a conventional VCSEL and inverse-cavity structures based on AlGaAs-compound multilayer system as shown in Fig. 3a. In the inverse-cavity structure, the top distributed-Bragg-reflection (DBR) multilayer in the conventional VCSEL structure is replaced by a GaAs guided-mode-resonance (GMR) reflector that provides desired amplified feedback towards the AlAs passive cavity. Considering emission wavelength around 800 nm from GaAs as a gain medium, we assume a trial case with parameters indicated in the figure caption. We include complex refractive index 2.95 + *i*1.6 × 10^−4^ for the AlAs passive cavity and 3.6 + *iκ* for the GaAs GMR reflector layer, where extinction coefficient *κ* yields net material gain constant *G*_0_ = –(4π/*λ*)*κ* with *λ* being wavelength of light in vacuum. We use the rigorous coupled-wave analysis (RCWA)^18^ for numerical calculation.

In Fig. 3b, we show normal-incidence reflectance spectrum of the inverse-cavity structure on wavelength (*λ*) and cavity length (*L*) domain that reveals cavity-resonance condition under the influence of the resonant reflection from the GMR reflector layer for *G*_0_ = 0. The cavity resonances create periodic dark minima labeled by order *m* indicating the number of field oscillation during one intracavity roundtrip as in the canonical Fabry-Pérot (FP) resonators. The resonant internal reflection from the GMR layer leads to two characteristic features in these cavity resonance minima. One is the FP order shift from *m* to *m* + 1. This is caused by resonant 2π-phase change in the internal reflection over a narrow GMR band from 800.2 to 800.8 nm. The second characteristic feature is vanishingly narrow resonance linewidth in the middle of the order shift region, *i.e.*, cavity resonances at *λ* = 800.476 nm. This is a bound state in the continuum (BIC) appearing as radiative decay towards the air cover vanishes at the internal reflectance maximum (*R*_max_ = 1) condition.

As *G*_0_ in the GMR layer increases from zero, the internal reflection is amplified (*R*_max_ > 1) as shown in Fig. 3c and the BIC features for different FP orders at *λ* = 800.476 nm can oscillate as lasing modes presumably with a minimal threshold gain constant. Considering attenuation *α* = 25.12 cm^−1^ inside the AlAs cavity and DBR reflectance *R*_0_ = 0.9899, radiative decay rate *α*_rad_ = 70.6 cm^−1^ of the GMR, and GMR’s radiative decay probability *η*_1_ ≈ 0.6 toward the AlAs cavity in our specific case here, we estimate an inverse-cavity threshold gain constant *G*_0_ = *G*_IC_ ≈ 0.46 cm^−1^ for the lowest-order BIC mode at *λ* = 800.476 nm and *L* = 224 nm from Eqs. (2) and (3) in an ideal case for *G*_0_ = *G*_B_–*α*_B_. Remarkably, Eq. (1) for the conventional VCSEL structure with the same cavity length yields a threshold gain constant *G*_0_ = *G*_conv_ ≈ 474 cm^−1^, which is remarkably higher from *G*_IC_ by a factor around 10^3^.

In order to better substantiate this intriguing property, we numerically calculate threshold gain constants *G*_IC_ and *G*_conv_ by using a *G*_0_-dependent resonance-excitation spectrum analysis, as provided in Supplementary Note II. Therein, we determine the threshold gain constant to be a *G*_0_ value for a scattering-matrix pole at which bandwidth of the resonance-excitation spectrum vanishes and its peak value diverges to the infinity. This frequency-domain analysis has been used to estimate ideal threshold gain constant that compensates necessary cavity losses^19,20^. However, it does not account for material dispersion and gain saturation nonlinearity and thereby cannot predict linewidth and intensity beyond the threshold condition. In spite of such limitations, the frequency-domain analysis provides a reasonable comparison for the fundamentally required threshold gain constant which is our major interest here.

In Fig. 3d, we show the result obtained as a function of cavity optical path length *nL*, where *n* is real-part refractive index of the cavity medium. In this result, *G*_IC_ for the inverse-cavity structure decreases for smaller *L* in contrast to increasing *G*_conv_ for the conventional VCSEL structure, as predicted in the previous section. For the lowest-order inverse-cavity mode at *L* = 224 nm as the shortest laser cavity, *G*_IC_ = 0.48 cm^−1^ ≈ 1.6 × 10^−3^·*G*_conv_. Notably, *G*_IC_ is also considerably lower than *G*_GMR_ = *α*_rad_ = 70.6 cm^−1^ ≈ 1.5 × 10^2^·*G*_IC_, which is threshold gain constant for the GMR reflector itself as a second-order distributed-feedback (DFB) laser in the absence of the bottom DBR.

In further consideration on the property *G*_IC_ <  < *G*_GMR_, *G*_IC_ is essentially lower than *G*_GMR_ because *G*_IC_ is reached at a condition for an amplified reflectance at a certain finite level while *G*_GMR_ is obtained when the amplified reflectance tends to the infinity as a pole in the scattering matrix of the GMR reflector itself. Looking into this aspect in connection with Eqs. (2) and (3), we derive an expression for threshold gain-constant ratio

5 $$M \equiv \frac{{G_{{{\text{IC}}}} }}{{G_{{{\text{GMR}}}} }} = \frac{{G_{{\text{B}}}^{{\text{(IC)}}} - \alpha_{{\text{B}}} }}{{G_{{\text{B}}}^{{\text{(GMR)}}} - \alpha_{{\text{B}}} }} = \frac{1}{1 + \xi }.$$

Here, *ξ* = 2*C*_2_[{*C*_1_^2^ + (4*αL*_c_)*C*_2_}^1/2^ − *C*_1_]^−1^. See Eq. (S3) in Supplementary Note I for expressions of coefficients *C*_1_ and *C*_2_. Importantly, *ξ* is always a positive number and *M* < 1. This implies that if *G*_GMR_ is substantially reduced down by additional optimization for a better DFB laser property, inclusion of it as an amplified reflector for an inverse-cavity laser necessarily results in a lower threshold gain *G*_IC_ than *G*_GMR_. Therefore, our trial case analysis clearly demonstrates the desired advantage of the inverse-cavity laser concept over the conventional systems such as VCSEL and second-order DFB laser structures.

Although the low threshold gain-constant property takes many advantages in laser operation, it is important to estimate lateral footprint size of the cavity and consequent net pumping power requirement. Comparing the proposed inverse-cavity and conventional VCSEL designs in Fig. 3, we calculate angular full-width at half maximum of the cavity modes for both structures and estimate their corresponding diffraction-limited beam sizes as the minimally possible footprint size *W*_min_ of the cavity. We obtain *W*_IC_ = 2.5 mm for *W*_min_ in the inverse-cavity design and *W*_conv_ = 13.5 μm for *W*_min_ in the conventional VCSEL design. Threshold pumping power ratio is then roughly estimated as (*W*_IC_/*W*_conv_)^2^⋅(*G*_IC_/*G*_conv_) ≈ 55, implying that the proposed inverse cavity strucrture requires significantly higher pumping power as a result of the inevitably large footprint size. Therefore, additional footprint-size reduction scheme should be introduced in order to take full advantages of the low threshold gain constant property.

There are several available methods for reducing lateral size of GMR reflector structures without substantial loss in the feedback efficiency^21^. They are mainly based on additional first-order distributed feedback gratings^22^ on the side edges and doubly-periodic GMR designs^23^. For example, we conduct a trial numerical analysis for an inverse-cavity configuration based on a doubly-periodic GMR design, as explained in Supplementary Note III. Therein, we obtain *G*_IC_ = 1.33 cm^−1^ ≈ 2.9 × 10^−3^·*G*_conv_ and *W*_IC_ = 172.7 μm ≈ 12.8·*W*_conv_. Threshold pumping power ratio is then roughly estimated as (*W*_IC_/*W*_conv_)^2^⋅(*G*_IC_/*G*_conv_) ≈ 0.47, implying that the threshold pumping power is a half of that for the conventional VCSEL structure. According to Ref. 21, the minimum footprint size of doubly periodic GMR reflectors is in the order of 10 × 10 μm^2^ and this corresponds to a typical footprint size of conventional VCSEL structures. Therefore, the low threshold property of an inverse-cavity structure should be sustainable without significant increase in the footprint size or threshold pumping power if a certain optimized size-reduction scheme is introduced in the resonant reflector design.

**Figure 4:**
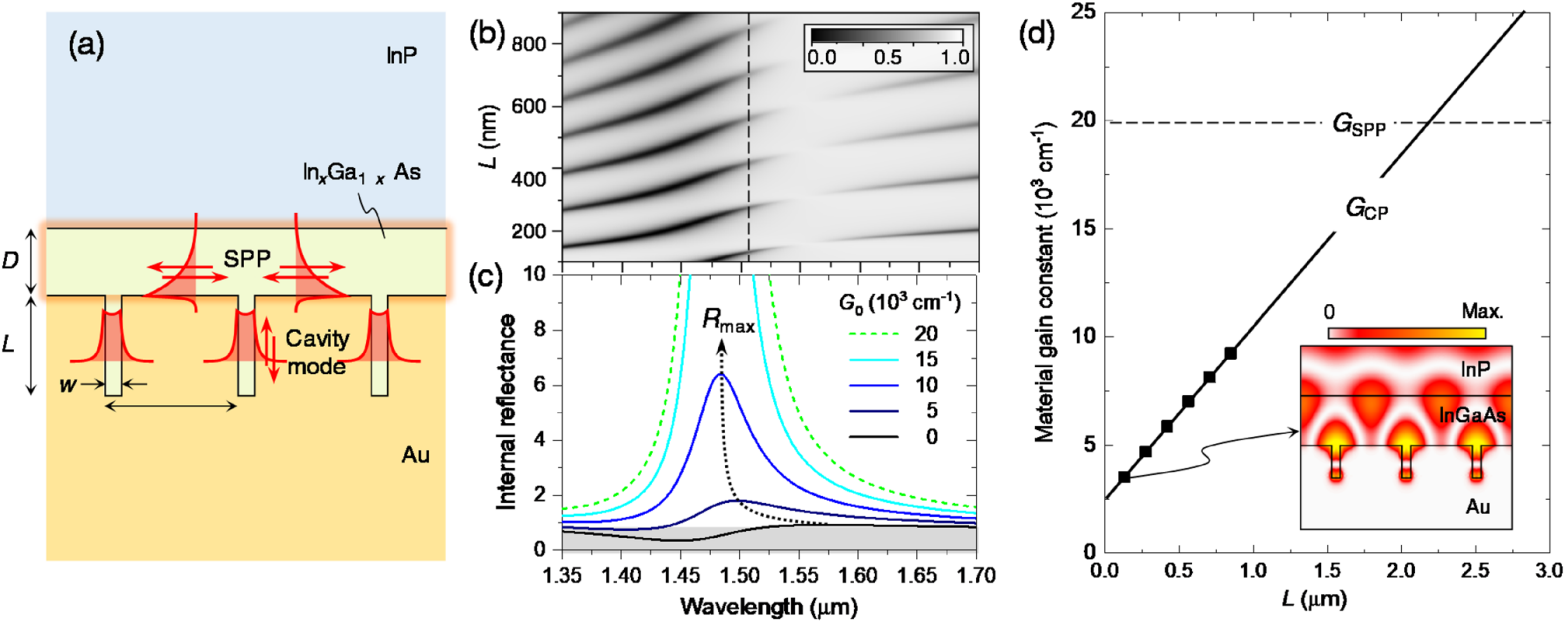
Plasmonic inverse-cavity laser property. (**a**) Structure geometry of an Au-InGaAs-based plasmonic inverse-cavity structure where periodic nanoslits function as plasmonic nanocavities and resonant grating for SPPs on the Au-InGaAs interface. (**b**) Reflectance spectrum on *L*-*λ* plane for a plasmonic inverse-cavity structure under normal incidence from the InP cover. Geometrical parameters *Λ* = 410 nm, *w* = 50 nm, *D* = 200 nm. We take frequency-dispersive optical constants of InP^25^, InGaAs^26^, and Au^27^ from the literature in consideration of relatively wide spectral range of the plasmonic resonance effects in our present case. (**c**) Amplified internal reflectance spectra due to a gain-assisted SPP resonance for increasing *G*_0_. (**d**) Calculated threshold gain constants *G*_CP_ for the plasmonic inverse-cavity structure as functions of cavity length *L*. *G*_SPP_ is threshold gain constant for the SPP resonance on the Au-InGaAs interface for open cavities with *L* = ∞.

We further investigate feasibility of the inverse-cavity structure for plasmonic lasers which potentially enable extremely small lasers taking advantages of deep subwavelength confinement and ultrafast dynamic properties^24^. We consider a periodic array of metal–insulator-metal (MIM) nanocavities in an Au-InGaAs-InP system as shown in Fig. 4a. In this specific geometry, the nano-slit cavities support FP-like resonances of metal–insulator-metal plasmonic guided modes, *i.e.*, cavity modes, and the Au-InGaAs interface accommodates surface-plasmon polaritons (SPP) which can be amplified by the stimulated SPP emission from coherent carrier recombination in the InGaAs layer. Hence, the internal reflection of the cavity plasmonic modes can be amplified by mediation of amplified SPPs under certain appropriate resonant-coupling conditions similar to the inverse-cavity VCSEL structure in Fig. 3.

Considering optical emission around *λ* = 1550 nm from the InGaAs layer, we take geometrical parameters as indicated in the caption of Fig. 4. We assume material gain constant *G*_0_ = − (4π/*λ*)*κ* only in the InGaAs layer between Au-InGaAs and InGaAs-InP interfaces in order to exclude direct amplification of the cavity-plasmonic modes in this proof-of-concept numerical analysis. In Fig. 4b, we reveal cavity-plasmonic resonance conditions from reflectance spectrum of the structure for *G*_0_ = 0 under normal light incidence from the InP cover. The periodic resonance dips are due to FP-like resonances of the cavity-plasmonic modes. In the similar manner to the previous VCSEL case, these cavity-plasmonic resonances also include the FP-order shift and BIC states around 1550 nm in response to the resonant internal reflection with SPP excitation in the second-order Bragg reflection regime.

Amplified internal-reflection spectrum from the gain-assisted SPP resonance is provided in Fig. 4c as a function of *G*_0_. For *G*_0_ = 0, the characteristically asymmetric Fano-resonance profile has a minimum at *λ* = 1440 nm and maximum *R*_max_ ≈ 1 at *λ* = 1550 nm where the BIC features in Fig. 4b appear at. As *G*_0_ increases, *R*_max_ is amplified from 1 and its spectral location shifts towards the SPP-resonance excitation center at *λ* = 1480 nm. For *G*_0_ = 2 × 10^4^ cm^−1^, *R*_max_ diverges to the infinity and we take this value as threshold gain constant *G*_SPP_ for the SPP as a second-order DFB lasing mode in our specific case here.

The blue shift of the *R*_max_ wavelength with increasing *G*_0_ pushes the lowest-gain inverse-cavity lasing wavelength for the cavity-plasmonic modes towards slightly shorter wavelength from the BIC point at *λ* = 1550 nm. Hence, we select *λ* = 1510 nm (dashed line in Fig. 4b) as an approximate optimal point and numerically determine threshold gain constant *G*_CP_ for each cavity-plasmonic FP-like resonance feature by using the *G*_0_-dependent resonance-excitation spectrum analysis as used in the previous VCSEL case. The result is provided in Fig. 4d. Calculated *G*_CP_ shows a desired linear dependence on cavity length *L* as expected for inverse-cavity laser structures in general. For the lowest FP order at *L* = 130 nm, *G*_CP_ = 3.52 × 10^3^ cm^−1^, which is only about 1/6 of *G*_SPP_ in spite of stronger absorption losses in the plasmonic cavity mode. Moreover, *G*_CP_ is only 1/5.5 of a threshold gain constant 1.93 × 10^4^ cm^−1^ for direct amplification of a plasmonic cavity mode at the lowest FP order in the absence of the gain-assisted SPP resonance. Therefore, the inverse-cavity approach can create remarkably efficient plasmonic lasers under appropriate design schemes.

We note that lasing modes in an inverse-cavity structure are in a hybrid nature because cavity modes essentially excites a resonant mode associated with the amplified reflection. For example, the inset of Fig. 4d shows magnetic field intensity distribution for the lowest FP-order cavity-plasmonic resonance at *L* = 130 nm. We see that a standing SPP mode at the Au-InGaAs interface placing its anti-node at the cavity mouth is strongly excited along with the FP-like cavity-plasmonic mode resonance. Accordingly, this type of laser may not fully exploit strong cavity-QED effects for deep-subwavelength plasmonic modes. Nevertheless, careful trade-off of this restriction with the advantage of low threshold lasing may yield optimal designs required for specific applications.

## Discussion

In conclusion, we have numerically demonstrated the inverse-cavity laser structures for their unusually low threshold-gain property in the shortest cavity regime. The proposed inverse-cavity amplification mechanism yields thousand times lower threshold gain in a conceivable VCSEL structure than the conventional approach and reduces threshold gain of a lattice plasmonic nanocavity laser by almost one order of magnitude.

In our proposed inverse-cavity structure, a key essential component is the amplified-feedback mechanism and it is obtainable with a gain medium involving nanophotonic resonance structures. Although we have treated strictly periodic structures in this proof-of-concept analysis, the proposed approach is in principle applicable for non-periodic cases where various beam control schemes can be included on demand. For example, in VCSEL structures, cross-sectional beam intensity, phase, and polarization profiles can be engineered by appropriately tapering geometrical parameters of a GMR reflector design to produce desired distributions such as super-Gaussian flat-top beams^28^, long depth-of-focus Bessel beams^29^, polarization vector beams^30^, orbital angular-momentum modes^31^, and many others. Therefore, it is of our great interest to derive and realize various periodic and non-periotic inverse-cavity laser designs taking advantages of low threshold gain and beam control capabilities.

## Supplementary Information

Supplementary Information 1.

Supplementary Information 2.

## Data Availability

All data generated or analyzed during this study are included in this published article (and its supplementary information files SI).
