# Supplementary material for: Inverse-cavity structure for low-threshold miniature lasers
Source: Sci Rep. 2022 Jul 5;12:11333. doi: 10.1038/s41598-022-15319-y (PMC9256698; doi:10.1038/s41598-022-15319-y)
Supplement: Supplementary file 2 — Supplementary Information 2. [file 41598_2022_15319_MOESM2_ESM.docx]

**Supplementary Information for**

**Inverse-cavity structure for low-threshold miniature lasers**

Gunpyo Kim, Seok Ho Song, and Jae Woong Yoon^†^

*Department of Physics, Hanyang University, Seoul 04763, Korea*

^†^Correspondence should be addressed to J.W.Y at [yoonjw@hanyang.ac.kr](mailto:yoonjw@hanyang.ac.kr)

**Supplementary Note I: Amplified reflectance by a nanophotonic resonance**

In a two-channel Fano-resonance problem, a generic temporal coupled-mode theory [17] describes reflection coefficient *r*_1_ as a superposition of non-resonant reflectivity *r*_D_ and resonant reflectivity *r*_R_ such that

, (S1)

where *ω*_0_ is resonance-center angular frequency, *α*_1_ is partial rate of the radiation decay toward the reflection channel, *α*_G_ = *G*_B_–*α*_B_ is partial rate of the gain-assisted amplification, *α*_rad_ is partial rate of the radiation decay, and *φ*_R_ is phase constant for the resonant reflectivity.

Resonant reflectivity *r*_R_ in the second term on the right-hand side of Eq. (S1) can be alternatively expressed by *r*_R_ = *η*_1_(1+*η*_G_)[1+*e^i^*^2^*^θ^*(*^ω^*)]*e^iφ^*^R^ with phase argument *θ*(*ω*) = tan^−1^[(*ω*−*ω*_0_)/(*α*_rad_−*α*_G_)], probability of the radiative decay toward the reflection channel*η*_1_ = *α*_1_/*α*_rad_, and ratio of the gain-assisted amplification rate to the total decay rate *η*_G_ = *α*_G_/(*α*_rad_−*α*_G_). This expression is conveniently visualized on a complex plane as shown in Fig. S1. The blue circle represents reflection coefficient *r*_1_^(0)^ for *α*_G_ = 0 (no gain) and it is characterized by radius *η*_1_ and center position *z*_c_^(0)^ such that the circle inscribes the unit circle (gray dotted) as a result of fundamental restrictions from the time-reversibility, reciprocity, and energy conservation in the elementary coupling processes [17]. The red circle represents reflection coefficient *r*_1_ for *α*_G_ > 0 and it is characterized by increased radius *η*_1_ = *η*_1_(1+*η*_G_) and center location *z*_c_ shifted to the right along the line connecting *r*_D_ and *z*_c_^(0)^. From this geometric representation, we can conveniently find an approximate expression of peak reflectance *R*_max_ = |*r*_max_|^2^ for *α*_G_ > 0 as

(S2)

**Figure S1. Geographical representation of amplified-reflection coefficient *r*_1_.** Parametric trace of *r*_1_(*ω*) for variable *ω* around resonance center *ω*_0_ appears as a circle with center *z*_C_ and radius *η*_1_(1+*η*_G_) on a complex plane. *r*_1_ describes the reflection coefficient in the presence of non-zero net gain (*α*_G_ > 0) while *r*_1_^(0)^ is the reflection coefficient in the absence of net gain or loss (*α*_G_ = 0). *r*_max_ corresponds to a length between the origin and the farthest point in the red circle.

where the coefficients are given by

and . (S3)

Here, Δ*φ* = arg(*r*_D_)–*φ*_R_ is the phase difference between *r*_D_ and *r*_R_ at *ω* = *ω*_0_ and we use a relation |*r*_max_^(0)^| = |*z*_c_^(0)^|+*η*_1_ = 1 for *α*_G_ = 0. Applying the definition of the parameters*η*_G_ = *α*_G_/(*α*_rad_–*α*_G_) and *α*_G_ = *G*_B_–*α*_B_ to Eq. (S2) immediately results in Eq. (3) in the main text.

**Supplementary Note II: Numerical determination of threshold gain constant**

Using the rigorous coupled-wave analysis, we calculate *G*_0_-dependent resonance-excitation spectra for both inverse-cavity resonances and guided-mode resonances (GMRs), as shown in Fig. S2. Under normal incidence and transverse electric polarization, we obtain the spectral responses for a GMR-assisted inverse-cavity structure and a GMR structure without the bottom distributed-Bragg-reflection layer, as schematically indicated in the insets of Figs. S2(a) and S2(c). As material gain constant *G*_0_ in the GaAs layer gradually approaches threshold gain constants *G*_IC_ and *G*_GMR_ for the inverse-cavity and GMR structures, respectively, spectral linewidths approach zero while field intensity associated with the resonance mode diverges to infinite, as shown in Figs. S2(b) and S2(d).

**Figure S2. G_0_-dependent resonance-excitation spectrum analysis. (a,b)** G_0_-dependent spectral profiles of the normalized cavity field intensity **(a)**, spectral linewidths, and peak cavity field intensity **(b)** for a fundamental inverse-cavity resonance. In **(a)**, the wavelength range of spectral profiles spans 100 fm at center of wavelength 800.475846 nm. Cavity field intensities are calculated from the electric field intensities between a GMR reflector and a bottom DBR mirror (Inlet schematic illustration in **(a)**). **(c,d)** For guided-mode resonances (GMRs), G_0_-dependent spectral profiles of the normalized GMR field intensity **(c)**, spectral linewidths, and peak cavity field intensity **(d)**. GMR field intensities are obtained from the electric field intensities of the GMR structure which is far from the bottom DBR mirror (Inlet schematic illustration in **(c)**). Q_0_ represents a maximum value of cavity or GMR field intensity without net modal-gain constant. *E*_0_ represents an electric field amplitude of an incident wave.

**Supplementary Note III: Inverse-cavity configuration applying a doubly periodic GMR element**

Considering low power consumption for lasing, we present another amplified feedback element using a doubly periodic resonant grating. This doubly periodic resonant gratings have large tolerance in angular spectrum so that finite size-reduction scheme can be included without significant linewidth broadening [1]. Figure S3(a) describes an inverse-cavity configuration with a doubly periodic resonant grating as an amplified feedback mechanism. Structural parameters this structure is included in the figure caption. Except for the top doubly periodic GMR reflector, the basic configuration including a bottom DBR mirror and passive AlAs cavity is identical to the inverse-cavity structure in Fig. 3(a) in the main text. The doubly periodic GMR reflector consists of a GaAs film.

In Fig. S3(b), we provide the resonance spectrum of this inverse cavity structure depending on the cavity length *L* in the absence of optical gain in the resonant reflector and it shows BIC features at *λ*_0_ = 800.87 nm for *m* = 1 and *λ*_1_ = 800.83 nm for *m* ≥ 2. A slight red-shift of the BIC feature for *m* = 1 is due to the effect of the DBR layers on the guided mode in the resonant reflector. Figure 3(c) shows that the internal reflection is amplified near λ_1_, as *G*_0_ increases. Using the *G*_0_-dependent resonance-excitation spectrum analysis, we find threshold the gain constant as explained in the main text. The result is shown in Fig. S3(d). Threshold gain constant for the fundamental cavity mode is only 1.33 cm^−1^. This value is 2.9×10^−3^ times lower than that of the conventional VCSEL structure in Fig. 3.

Considering plane-wave spectrum occupying most of the energy in the resonant element, we estimate minimally possible horizontal size *W*_min_ of the cavity structure. We analyze angular dependence of internal reflectance spectrum for the top GMR reflector and calculate angular full-width at half maximum Δ*θ* = 0.18 ° of the fundamental cavity mode at resonant wavelength λ_0_. Following the standard diffraction theory, we estimate *W*_IC_ = 2*λ*_0_(*n*Δ*θ*)^−1^ = 172.7 μm as the minimally possible footprint length determined by a diffraction-limited spot size [2].

**Figure S3. Doubly periodic GMR-assisted inverse-cavity structure.** (a) Geometry of an inverse-gain laser cavity oscillator with a doubly periodic grating. Amplified feedback toward the passive cavity is obtained from a GaAs-based GMR within the doubly periodic grating. (b) Reflectance spectrum on *L*-*λ* plane for an inverse-cavity structure under normal incidence from air cover. Structural parameters of the bottom DBR mirror and optical constants correspond to those in figure 3(a). For the doubly periodic grating, geometrical parameters are *D* = 250 nm, *d* = 250 nm, *Λ* = 231.3 nm, *w*_1_ = 0.29*Λ*, *w*_2_ = 0.25*Λ*, and *w*_3_ = 0.21*Λ*. (c) Amplified internal reflectance spectra by a gain-assisted GMR for increasing *G*_0_. (d) Calculated threshold gain constants *G*_IC_′ for the inverse-cavity configuration and *G*_conv_ for the conventional VCSEL structure as functions of cavity optical path length *nL*.

**References**

[1] Mizutani, A., Kikuta, H., & Iwata, K., Wave localization of doubly periodic guided-mode resonant grating filters, *Opt. Rev.* **10**, 13-18 (2003).

[2] Bendickson, J. M. et al. Guided-mode resonant subwavelength gratings: effects of finite beams and finite gratings. *JOSA A* **18**, 1912-1928 (2001).
